# Interfacial and Bulk Stabilization of Oil/Water System: A Novel Synergistic Approach

**DOI:** 10.3390/nano10020356

**Published:** 2020-02-18

**Authors:** Ahmad Shakeel, Ujala Farooq, Claire Chassagne

**Affiliations:** 1 Faculty of Civil Engineering and Geosciences, Department of Hydraulic Engineering, Delft University of Technology, Stevinweg 1, 2628 CN Delft, The Netherlands; C.Chassagne@tudelft.nl; 2 Faculty of Aerospace Engineering, Aerospace Manufacturing Technologies, Delft University of Technology, Kluyverweg 1, 2629 HS Delft, The Netherlands; U.Farooq@tudelft.nl

**Keywords:** synergistic stabilization, nanoparticles, bigels/bijels, rheology

## Abstract

Oil/water emulsions are usually stabilized either by interfacial modification using nanoparticles and surfactants (stated as pickering emulsion or bijels) or by bulk stabilization with the help of low-molecular-weight or polymeric gelators (known as bigels) in response to some external stimuli (e.g., pH, temperature). Both these approaches result in different systems that are quite useful for different applications, including catalysis, pharmaceutical and agrochemicals. However, these systems also possess some inherent drawbacks that need to be addressed, like difficulty in fabrication and ensuring the permanent binding of nanoparticles at the oil/water interface, in case of nanoparticles stabilized emulsions (i.e., interfacial stabilization). Similarly, the long-term stability of the oil/water systems produced by using (hydro/organo) gelators (i.e., bulk stabilization) is a major concern. Here, we show that the oil/water system with improved mechanical and structural properties can be prepared with the synergistic effect of interfacial and bulk stabilization. We achieve this by using nanoparticles to stabilize the oil/water interface and polymeric gelators to stabilize the bulk phases (oil and water). Furthermore, the proposed strategy is extremely adaptable, as the properties of the resultant system can be finely tuned by manipulating different parameters such as nanoparticles content and their surface functionalization, solvent type and its volume fraction, and type and amount of polymeric gelators.

## 1. Introduction

The mixture of oil and water is typically unstable, particularly in the absence of any additive/stabilizer, due to its polarity difference. However, from a commercial point of view, this mixture is of crucial importance due to the exciting properties of both phases. There are two well-known strategies that exist to tackle this instability problem. First one deals with the modification of the oil/water interface with the help of nanoparticles or surfactants or a combination of both [1,2,3,4,5,6]. The second approach targets the bulk stabilization of one or both phases by adding low-molecular-weight (LMW) or polymeric gelators [7,8,9], to obtain the stable oil/water emulsion gel.

The interfacial stabilization of the oil/water system, with the help of nanoparticles, has extensively been studied in the literature [10,11,12,13]. It usually results in two types of morphologies, depending upon the volume fraction of each phase. If one phase is dispersed within the other, the interfacially stabilized oil/water system is then referred to as pickering emulsion. However, if the two phases de-mix by spinodal decomposition and form a bi-continuous morphology, the adsorption of nanoparticles at oil/water interface during de-mixing results in bi-continuous jammed emulsions known as bijels. Nanoparticles are known to adsorb at the oil/water interface very rapidly and stabilize a higher surface area for a given volume fraction. However, permanent binding and maximum uptake of nanoparticles to the oil/water interface has been demonstrated to be difficult, to stabilize the whole system [14].

The second technique to stabilize the oil/water emulsion involves the formation of a 3D network of LMW or polymeric gelators in each phase (i.e., gelation), before or after mixing the phases, and the resultant system is usually known as bigel [15,16,17]. Different morphologies can be obtained using this approach as well, by varying the volume fraction of each phase, such as oil-in-water, water-in-oil, or bi-continuous type. However, these systems can undergo phase separation due to the destruction of 3D polymeric network by shearing action.

Quite recently, a study has been reported on the stabilization of the oil/water system to form bijel using mixture of nanoparticles and polymers [4]. The authors reported the adsorption of polymer-modified nanoparticles at the oil/water interface and the stabilization of the system far from the de-mixing point, having an interfacial tension of the order of 20 mN m^−1^. These interesting results provide the motivation to combine the usefulness of nanoparticles and polymers in a single system. Therefore, in this study, the interfacial and bulk stabilization of the oil/water system have been achieved by using a mixture of silica nanoparticles (i.e., interfacial stability) and polymeric gelators (i.e., bulk stabilization). According to the author’s knowledge, this is the first attempt to stabilize the oil/water emulsion by having both interfacial and bulk modification.

## 2. Results and Discussion

**Figure 1:**
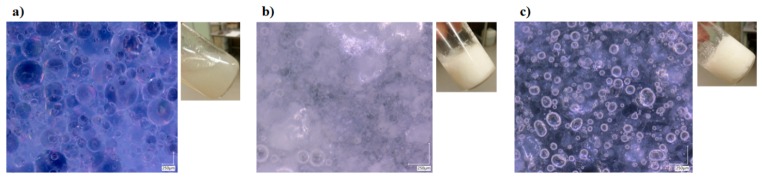
Micrographs of (**a**) pickering emulsion, (**b**) bigel, and (**c**) NPs bigel.

In this study, different systems were prepared and analysed to verify the synergistic enhancement in the properties of the proposed system (i.e., both interfacial and bulk stabilization). First of all, a typical pickering emulsion (stated as pickering) was prepared by stabilizing equal amount of oil in water (i.e., liquid paraffin in water) with the help of silica nanoparticles. Figure 1a shows the stabilization of oil droplets with the help of nanoparticles, and also the liquid-like behavior of the resultant system. To further enhance the stability of the system, only water phase was jellified with the help of sodium salt of carboxymethyl cellulose (stated as HG pickering). Similarly, in another system, only oil phase was structured using Myverol (monoglycerides) and termed OG pickering. However, these systems were quite unstable, even for a very short period of time, because of the mismatch between the mechanical properties of each phase, as already reported in literature for emulsion gels (i.e., with one structured phase) [18]. To avoid this mismatch, both phases were structured using polymeric gelators (Myverol and sodium salt of carboxymethyl cellulose) without the addition of nanoparticles (termed as bigels). This system was fairly stable, and the micrograph shows the existence of structured domains along with the oil or water droplets (Figure 1b). Finally, the nanoparticles-based bigel system (stated as NPs bigels) was prepared to stabilize the interface and bulk of the oil/water system. In this case, polymeric gelators were first dissolved in each phase and then the nanoparticles were incorporated in the liquid oil/water mixture. The nanoparticles adsorbed at the oil/water interface and provide the interfacial stability along with a particular morphology. Afterwards, with the help of external stimuli (i.e., temperature reduction), the formation of 3D polymeric network (i.e., gelation) was achieved in each of the phase to have bulk stabilization. The micrograph of the prepared system shows the existence of a structured network along with the nanoparticle-stabilized droplets (Figure 1c), which verified the dual stabilization of the system.

**Figure 2:**
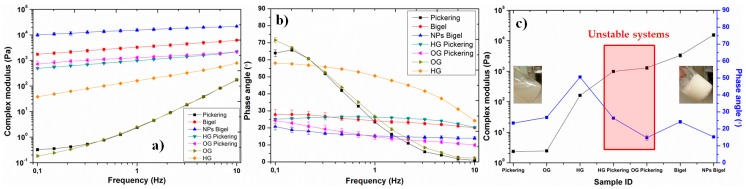
(**a**) Complex modulus and (**b**) phase angle as a function of frequency for different oil/water systems, and (**c**) values of complex modulus and phase angle at 1 Hz for different investigated samples (bars represent standard deviation).

Apart from microscopic analysis, rheological investigation was also carried out for the prepared systems, which is a useful tool to analyse the structure–property relationship in different systems [19]. Frequency sweep tests were performed, within the linear viscoelastic regime, for the samples to investigate their mechanical properties without disturbing the structure. In order to compare the properties of gelled systems, individual gels based on water phase (HG) and oil phase (OG) were also analysed. The results are presented in terms of complex modulus and phase angle as a function of frequency for different systems (Figure 2a–c). Three systems (pickering emulsion, hydrogel, and organogel) showed liquid-like behavior with smaller complex modulus values, higher phase angle values, and strong dependence of these properties on the frequency. On the other hand, remaining samples displayed solid-like characteristics with higher modulus and lower phase angle values. It is clearly evident that the incorporation of nanoparticles (i.e., interfacial stabilization) into the bigels resulted in better mechanical properties, see Figure 2c. This analysis also verifies the synergistic enhancement in the properties of the dual stabilized oil/water system. However, polymeric chains can also stabilize the interface or can modify the surface properties of nanoparticles, which needs further investigation.

**Figure 3:**
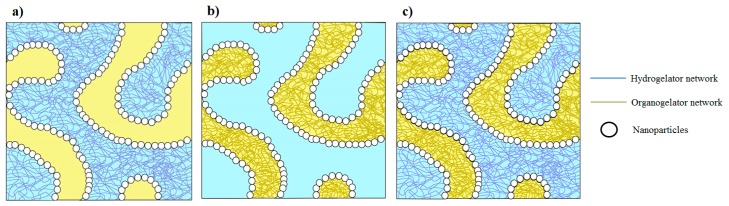
Proposed schematics of bijel systems with (**a**) structured water phase, (**b**) structured oil phase, and (**c**) both structured phases.

In comparison with pickering emulsions or bigels, we can readily prepare stabilized oil/water systems with better structural and mechanical properties using mixture of nanoparticles and organo/hydro gelators. In literature, it is already shown that the properties of bigels can be manipulated by playing with the gelator amount and volume fraction of each phase [20]. This shows that the properties of the proposed system can further be enhanced by varying the amount of structuring agents and the volume fraction of each phase. Furthermore, it is also possible to play with the particle surface functionality, particle shape, particle concentration, oil type, and gelator type. The proposed strategy can also be quite useful to prepare bijels (instead of pickering emulsions) with the structured phase(s) (see Figure 3). This suggests the potential of producing systems with properties covering a wide range of applications, including food, cosmetics, and pharmaceutical. These formulations can also be a potential and sustainable candidate for agrochemical applications by exploiting the ability of nanoparticles or the gel structure to capture and release the active pesticide molecules, as nano-emulsions are quite useful for the pests control [21].

## 3. Conclusions

In conclusion, interfacially and bulk stabilized oil/water systems have been prepared by adsorbing nanoparticles at the oil/water interface and by forming a 3D polymeric network in the bulk phases. We have shown that the advantages of the prepared system are manifold: easy to produce, better mechanical and structural properties, and possibilities of making diverse systems. The proposed system can be effectively used in food and cosmetics, because of the significant importance of oil/water emulsion and nanoparticles in these fields. Furthermore, we foresee the use of this system for drug delivery applications by controlling the mechanical properties of individual phases by external stimuli (i.e., pH or temperature) or by using stimuli-responsive nanoparticles.

## 4. Methods

### 4.1. Materials

Monoglyceride Myverol™ (kindly provided by Kerry Bioscience, Bristol, United Kingdom) was used as an organogelator to structure the oil phase. The liquid paraffin, glycerol (≥99.5%), and sodium salt of carboxymethylcellulose (i.e., used as hydrogelator) were purchased from Sigma-Aldrich, Steinheim, Germany. The silica nanoparticles were also obtained from Sigma-Aldrich, Steinheim, Germany with an average diameter of 12 nm and specific surface area of 175–225 m^2^/g. Deionized water (DI) was obtained from a Milli-Q water purification system (Merck Millipore, Darmstadt, Germany). All the chemicals were of analytical grade and used as received without any purification.

### 4.2. Preparation of Different Oil/Water Systems

First of all, the pickering emulsion was prepared by dispersing 0.25 g of silica nanoparticles into 5 mL of DI water at room temperature. The equal volume of oil phase was then added to the nanoparticles solution and vigorously mixed at 2000 rpm, resulting in nanoparticle-stabilized emulsion. In order to have the structured oil phase in this pickering emulsion, 0.5 g of Myverol was first dissolved into the oil phase at 80 °C until complete melting of the gelator. The resultant oil solution was then vigorously mixed with the aqueous nanoparticles solution at higher temperature (80 °C) to produce pickering emulsion, which was then cooled down at 20 °C to produce organogel based pickering emulsion (OG pickering). Similarly, the hydrogel-based pickering emulsion was produced by dispersing 0.3 g of sodium salt of carboxymethyl cellulose into 2 g of glycerol, in order to avoid the aggregation of polymer powder. The pickering emulsion (as prepared previously) was then added to this mixture at room temperature and stirred to produce the hydrogel based pickering emulsion. The synergistic stabilization (both interfacial and bulk) was achieved by dispersing the OG pickering emulsion (instead of pickering emulsion), before cooling down, to the mixture of hydrogelator and glycerol at room temperature with continuous stirring and resulting in a system termed ‘’NPs bigels’’. Hydrogel (HG), organogel (OG), and bigel were also prepared by the same procedure as already mentioned, without the addition of nanoparticles, in order to compare the properties of the proposed system with the conventional systems.

## 5. Characterization

Microscopic images were obtained using a digital microscope (VHX-5000, Keyence, Osaka, Japan) at 40 times magnification. Rheological analysis of prepared oil/water systems was performed using Haake MARS I Rheometer (Thermo Scientific, Karlsruhe, Germany) with a parallel plate geometry (diameter = 35 mm). Preliminary oscillatory stress sweep tests were performed to check the linear viscoelastic limit of the systems. After that, frequency sweep tests, within linear viscoelastic limit, were performed to investigate the rheological behavior of the systems.
